# ROCK inhibitor fasudil attenuated high glucose-induced MCP-1 and VCAM-1 expression and monocyte-endothelial cell adhesion

**DOI:** 10.1186/1475-2840-11-65

**Published:** 2012-06-13

**Authors:** Hailing Li, Wenhui Peng, Weixia Jian, Yuanmin Li, Qi Li, Weiming Li, Yawei Xu

**Affiliations:** 1 Department of Cardiology, Shanghai Tenth People’s Hospital, Tongji University, School of Medicine, Shanghai, China; 2 Department of Endocrinology, Xinhua Hospital, Shanghai Jiaotong University, School of Medicine, Shanghai, China

**Keywords:** High glucose, Fasudil, Monocyte chemoattractant protein-1, Vascular cell adhesion molecule-1

## Abstract

**Background:**

Previous studies suggested that the RhoA/ROCK pathway may contribute to vascular complications in diabetes. The present study was designed to investigate whether ROCK inhibitor fasudil could prevent high glucose-induced monocyte-endothelial cells adhesion, and whether this was related to fasudil effects on vascular endothelial cell expression of chemotactic factors, vascular cell adhesion molecule-1 (VCAM-1) and monocyte chemoattractant protein-1 (MCP-1).

**Methods:**

HUVECs were stimulated with high glucose (HG) or HG + fasudil in different concentration or different time. Monocyte-endothelial cell adhesion was determined using fluorescence-labeled monocytes. The mRNA and protein expression of VCAM-1 and MCP-1 were measured using real-time PCR and western blot. The protein levels of RhoA, ROCKI and p-MYPT were determined using western blot analysis. ELISA was employed to measure the expression of soluble VCAM-1 and MCP-1 in cell supernatants and human serum samples.

**Results:**

Fasudil significantly suppressed HG-induced adhesion of THP-1 to HUVECs. Fasudil reduced Rho/ROCK activity (as indicated by lower p-MYPT/MYPT ratio), and prevented HG induced increases in VCAM-1 and MCP-1 mRNA and protein levels. Fasudil also decreased MCP-1 concentration in HUVEC supernatants, but increased sVCAM-1 shedding into the media. In human diabetic subjects, 2 weeks of fasudil treatment significantly decreased serum MCP-1 level from 27.9 ± 10.6 pg/ml to 13.8 ± 7.0 pg/ml (*P* < 0.05), while sVCAM-1 increased from 23.2 ± 7.5 ng/ml to 39.7 ± 5.6 ng/ml after fasudil treatment (*P* < 0.05).

**Conclusions:**

Treatment with the Rho/ROCK pathway inhibitor fasudil attenuated HG-induced monocyte-endothelial cell adhesion, possibly by reducing endothelial expression of VCAM-1 and MCP-1. These results suggest inhibition of Rho/ROCK signaling may have therapeutic potential in preventing diabetes associated vascular inflammation and atherogenesis.

## Introduction

Macrovascular complications including atherosclerosis are the leading causes of morbidity and mortality in patients with diabetes mellitus [1]. The underlying mechanisms of vascular impairment in diabetes are not precisely defined. A potential contribution to atherogenesis in diabetes is increased adhesion of circulating monocytes to the vessel wall, which is believed to be exacerbated by hyperglycemia. Transmigration of monocytes into the subendothelial space and subsequent transformation into macrophage-derived foam cells are key events in atherogenesis [2,3]. These processes are partly regulated by chemotactic factors such as monocyte chemoattractant protein-1 (MCP-1) and endothelial vascular cell adhesion molecule-1 (VCAM-1) [4,5].

ROCK belongs to the AGC (PKA/PKG/PKC) family of serine/threonine kinases and is a major downstream effector of the small GTPase RhoA. Recent studies suggest that the RhoA/ROCK pathway may contribute to diabetic vascular complications [6-8]. The Rho/ROCK pathway has drawn considerable attention for its involvement in cardiovascular disease such as hypertension, vasospastic angina, ischemic stroke, heart failure [9,10], as well as atherosclerosis [11]. Blocking Rho kinase (ROCK) reduced both the early development and later progression of atherosclerotic plaques in apoE KO mice [12] and caused disappearance of coronary vasospastic activities in swine [13]. Rho/ROCK has also been implicated in the regulation of vascular tone and proliferation, as well as smooth muscle contraction, cell adhesion and motility [10]. There is evidence that inhibition of ROCKs causes activation of endothelial nitric oxide synthase (eNOS) and reduction of vascular inflammation [6,14]. However, the role of Rho/ROCK in glucose-induced monocyte-endothelial cells adhesion and migration is not known. Fasudil is the only ROCK inhibitor that has been employed clinically since 1995 [15]. Additionally, postmarketing surveillance studies reported that fasudil was considered reasonably safe and effective in humans [16].

Here we performed experiments to examine the effects of fasudil on high glucose induced monocyte adhesion to endothelial cells. Endothelial cell expression of adhesion and chemotactic factors VCAM-1 and MCP-1 was analyzed to explore potential mechanisms by which high glucose and the Rho/ROCK pathway regulates monocyte/endothelial cell interactions. In addition, the effect of fasudil on soluble vascular cell adhesion molecule-1 (sVCAM-1) and MCP-1 was examined in diabetic patients.

## Materials and methods

### Cells culture conditions

Human umbilical vein endothelial cells (HUVECs) were purchased from Clonetics Cell Discovery Systems (San Diego, CA, USA). HUVECs were routinely maintained at 37 in a humidified atmosphere of 95% air and 5% CO_2_ in DMEM (Gibco, NY, USA) supplemented with 10% fetal bovine serum (Gibco, NY, USA). For all experiments, HUVECs were used between passage 3 and 10. Human monocytic cell line THP-1 was obtained from Institute of Biochemistry and Cell Biology in Shanghai and was grown in RPMI 1640 medium (Gibco, NY, USA) supplemented with 2 mM L-glutamine and 10% fetal bovine serum.

After detachment of 90% confluent HUVECs from the flasks with 0.025% trypsin, the cells were plated in 6-well plates and stimulated with HG (20 mM) or HG (20 mM) + fasudil (10^-5^ mM) for the specified times (0 h, 3 h, 6 h, 12 h, 24 h, 48 h). In other experiments, HUVECs were subjected to six conditions: (1) 5.5 mM glucose as control; (2) 20 mM glucose; (3) 33 mM glucose; (4) 33 mM glucose + fasudil (10^-6^ mM); (5) 33 mM glucose + fasudil (10^-5^ mM); and (6) glucose (5.5 mM) + mannitol (14.5 mM). Total cellular RNA, protein and cell culture supernatants were collected for further study.

### Analysis of monocyte-endothelial cell adhesion

The cell adhesion assay was performed as described in a previous study [17] . HUVECs were grown to confluence in 6-well plates and treated with HG or HG + fasudil for 12 h or 24 h. THP-1 monocytes labeled with BCECF-AM was added to each well in the same conditions, for 1 hour. After incubation, the medium containing monocytes was aspirated and the monolayer was gently washed with PBS three times to remove the unbound monocytes. The fluorescence was measured at an excitation and emission wavelength of 488 nm and 535 nm by fluorescence microscopy (Leica DMI6000, Leica, Germany). The number of adherent cells was expressed as fluorescence intensity and the adhesion data are represented in terms of the fold change compared with the control values. Three fields were captured per experimental condition. Individual treatments were performed in duplicates, and the entire set of experiments was repeated three times.

### Total RNA extraction and real-time quantitative PCR

Total RNA was extracted using RNeasy Mini Kit (Qiagen, CA, USA) and 1 μg RNA was reverse transcribed to cDNA with PrimeScript® RT reagent Kit (Takara Bio inc., Otsu, Japan). Real-time PCR assays were performed using an Applied Biosystems 7900 (Applied Biosystems, Foster City, USA). The mRNA expression was determined using SYBR® Premix Ex Taq^TM^ (Takara Bio inc., Otsu, Japan). Samples were denatured at 95°C for 1 min, followed by 40 PCR cycles, each cycle consisting of 95°C for 5 s, 60°C for 1 min. The primers employed were as follows: VCAM-1: Sense 5' CCC TTG ACC GGC TGG AGA TT 3'; Antisense 5' TGG GGG CAA CAT TGA CAT AAA GTG 3'; MCP-1: Sense 5' CCC CAG TCA CCT GCT GTT AT 3'; Antisense 5' CCA CAA TGG TCT TGA AGA TCA C 3'; GAPDH: Sense 5' ACG GAT TTG GTC GTA TTG GG 3', Antisense 5' TGA TTT TGG AGGGAT CTC GC 3'. Human VCAM-1 and MCP-1 mRNA expressions were calculated with GAPDH as an internal control.

### Protein extraction and western blot analysis

Cells were lysed with ice-cold RIPA, centrifuged at 10,000 rpms for 5 minutes at 4 °C, and the supernatants were collected. Protein concentrations in the supernatants were measured using the BCA protein assay kit (Pierce Chemical Company, Rockford, USA). Cell homogenates (40 μg of protein) were separated on 8% SDS-polyacrylamide gel electrophoresis and transferred to PVDF, then washed with Tris-Buffered Saline, blocked with 5% skimmed milk powder except p-MYPT using 5% BSA in Tris-Buffered Saline Tween-20 for 1 hour, and incubated with the appropriate primary antibody at dilutions recommended by the supplier. Then the membrane was washed and primary antibodies were detected with secondary antibody conjugated to horseradish peroxidase for 1 h at room temperature. The blots were then developed with SuperSignal enhanced chemiluminescent substrate solution (Pierce Chemical Company, Rockford, USA). Anti-VCAM-1 and anti-MCP-1 antibody used in this study were purchased from Santa Cruz Biotechnology (Santa Cruz, CA, USA), anti-β-actin, anti-ROCKI, anti-RhoA, anti-p-MYPT and anti-MYPT antibodies were purchased from Cell Signaling Technology (Danvers, Massachusetts, USA).

### Clinical study

The clinical study protocol was approved by Ethics Committees of Shanghai Tenth Hospital and Xinhua Hospital. Patients with diabetes were enrolled in the program and all study participants were given written informed consents. Afterwards participants underwent a complete medical history and physical examination and then were divided into fasudil group and control group randomly. Diabetes was diagnosed according to the WHO criteria [18]. Exclusion criteria were current use of lipid lowering medication, inflammatory disease, deranged liver or renal function, and a major cardiovascular event within the last 3 months. Patients were randomized in a single-blind manner to receive either placebo or fasudil (Chase Sun Pharmaceutical Co., Ltd, TJ, China) 30 mg twice a day by intravenous drip for 30 minutes every day according to the protocol indicated. At admission and two weeks later, serum samples were collected after a 10-hour overnight fast and stored at −70°C.

### ELISA and biochemical investigations

Serum soluble VCAM-1 (sVCAM-1) and MCP-1 concentrations were measured with commercially available ELISA kits (BD Biosciences, CA, USA) according to the manufacturers’ instructions. The sensitivities of the sVCAM-1 and MCP-1 ELISA were 1 ng/ml and 4 pg/ml, respectively. Whereas the intra- and inter-assay precision in terms of percent coefficient of variance (CV%) for sVCAM-1 and MCP-1 ELISA were < 10% and < 15%, respectively. Serum levels of glucose and lipid profiles, including total cholesterol, low-density lipoprotein cholesterol, high-density lipoprotein cholesterol, etc. were measured by colorimetric enzymatic assay systems (Roche MODULAR P-800, Swiss Confederation) as indicated before [19].

### Statistical analysis

Continuous variables were described as means ± SD; Differences among multiple groups were analyzed by one-way ANOVA. Bonferroni multiple comparison was used for comparisons among multiple treatment groups and the control group. Paired and unpaired *t* test was used for comparisons the effects of fasudil on diabetic patients and control group. A 2-sided probability level of ≤ 0.05 was taken as significance. All analyses were done with SPSS for Windows 13.0 (SPSS Inc, Illinois, USA).

## Results

### Fasudil inhibited the HG-mediated monocyte-endothelial cells adhesion *in vitro*

**Figure 1:**
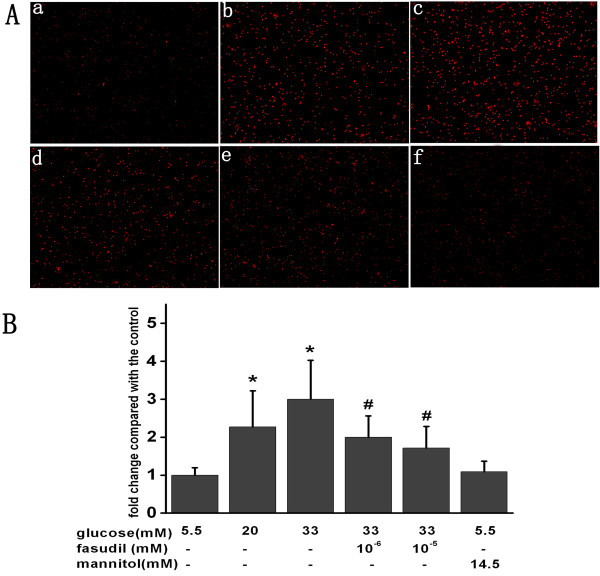
**Fasudil attenuated the adhesion of monocyte to HUVECs.A**: Confluent monolayers HUVECs were grown on 6-well plates and stimulated with HG or HG + fasudil for 24 hours. Subsequently, THP-1 labeled with BCECF-AM was added to each well in the same conditions. BCECF-AM fluorescent intensity of adherent cells were measured at excitation/emission wave length of 488/535 nm respectively. Results were expressed as BCECF-AM fluorescence fold induction compared with control. a control, b HG (20 mM), c HG (33 mM), d HG (33 mM) + Fasudil (10^-6^ mM), e HG (33 mM) + Fasudil (10^-5^ mM), f 14.5 mM mannitol. **B**: Means ± SD of at least three experiments are shown. * *P* < 0.05, when compared to control. # *P* < 0.05 when compared to HG (33 mM) alone. Mannitol was used as a control for osmolarity.

We used BCECF-AM labeled monocytes to evaluate the effect of fasudil on adhesion of monocytes to HUVECs. As shown in Figure 1, HG treatment of HUVECs for 24 h increased THP-1 cells adhesion as compared with control group (20 mM D-glucose increased adhesion 2.2 fold, while 33 mM D-glucose caused a 3.1-fold increase, both *P* < 0.05). A similar trend was observed within 12 hours (data not shown). Fasudil treatment significantly suppressed HG-induced adhesion of THP-1 cells, suggesting the HG induced increase in monocyte adhesion to endothelial cells is dependent upon Rho/ROCK activity. Similar results were obtained at 12 h (data not shown). As a control for osmolarity, mannitol had no effect on monocyte-endothelial cells adhesion.

### Fasudil attenuated HG-induced expression of VCAM-1 and MCP-1 in HUVECs

**Figure 2:**
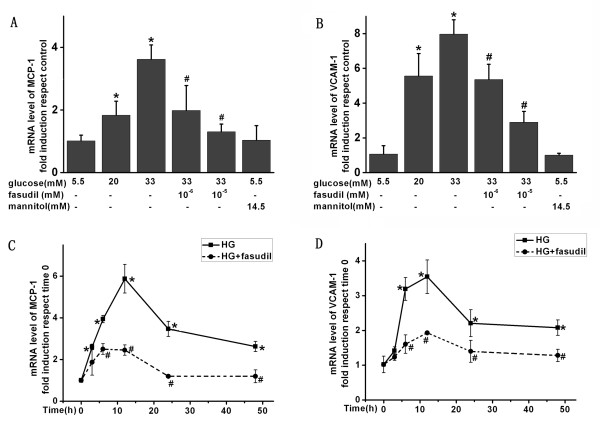
**Fasudil attenuated high glucose induced VCAM-1 and MCP-1 induction in HUVECS.** mRNA expression of VCAM-1 and MCP-1 genes in HUVECs was assessed by real-time PCR. The mRNA level of VCAM-1 and MCP-1 was normalized by GAPDH mRNA level. (A and B) Simultaneous treatment of HUVECs with fasudil for 24 h decreased HG-induced expression of MCP-1(**A**) and VCAM-1(**B**). A high concentration of fasudil (10^-5^ mM) had a more pronounced effect than the low concentration of fasudil (10^-6^ mM). Similar increased concentrations of mannitol did not affect VCAM-1 and MCP-1 expression. * *P* < 0.05 when compared to control. # *P* < 0.05 when compared to HG (33 mM) alone. (C and D) Time course of MCP-1(**C**) and VCAM-1(**D**) expression: HUVECs were cultured with medium containing 5.5 mM glucose, then the medium was changed to that with HG (20 mM, squares), or HG + fasudil(10^-5^ mM, circle) followed by further incubation for the indicated time. Results were expressed as fold induction respect time 0. Means ± SD of three experiments was shown. * *P* < 0.05 when compared to time 0, # *P* < 0.05 when compared to HG alone.

**Figure 3:**
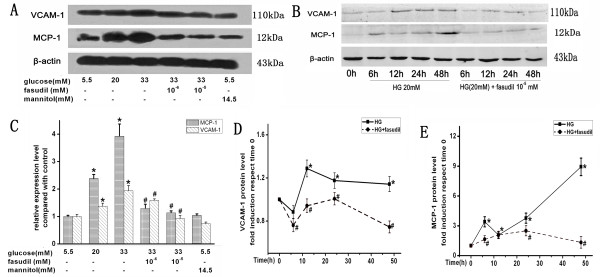
**Effects of HG and fasudil on VCAM-1 and MCP-1 protein in HUVECs.** VCAM-1 and MCP-1 protein levels were determined by Western blot analysis. Each band density was normalized by its own control. **A** demonstrates the dose-dependent effects of HG and fasudil. **B** is representative time-dependent effects of HG and fasudil. **C** is the summary of data (means ± SD) from the dose-dependent experiments. * *P* < 0.05 when compared to control. # *P* < 0.05 when compared to HG (33 mM) alone. **D** and **E** is the summary of data (means ± SD) from the time-dependent experiments. * *P* < 0.05 when compared to time 0, # *P* < 0.05 when compared to HG alone.

To understand the underlying molecular mechanism by which fasudil attenuates HG-induced monocyte-endothelial cells adhesion, we determined the effect of HG and fasudil on endothelial expression of adhesion and chemotactic factors VCAM-1 and MCP-1. Interestingly, HG (20 mM and 33 mM) for 24 h significantly increased HUVECs expression of VCAM-1 and MCP-1 mRNA (Figure 2) and protein (Figure 3). Extended treatment for 48 hours also significantly increased expression of both of these factors, particularly MCP-1. Importantly, fasudil treatment significantly attenuated the HG-induced increase in VCAM-1 and MCP-1 mRNA (Figure 2) and protein (Figure 3). This suggests fasudil may reduce monocyte adhesion to the endothelium by decreasing HG induced endothelial expression of these adhesion and chemotactic factors.

### Fasudil attenuated ROCK activity and ROCKI protein expression

**Figure 4:**
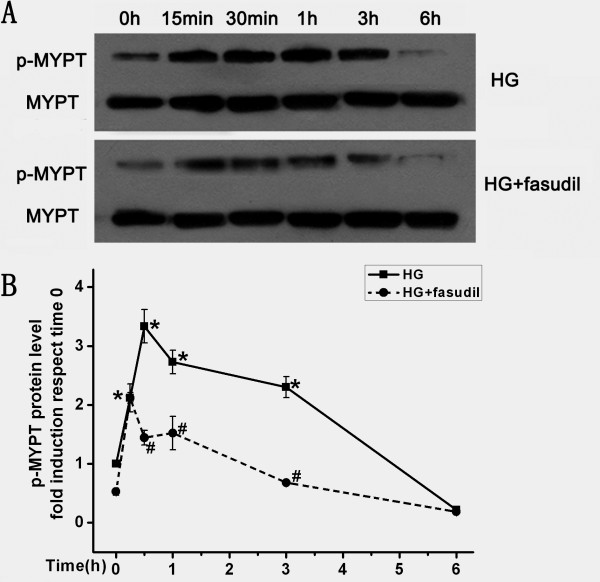
**Effect of fasudil on HG-induced Rho/rock activation.A**: Confluent HUVECs were stimulated with HG or HG (20 mM) + fasudil (10^-5^ mM) for different incubation times (0, 15 min, 30 min, 1 h, 3 h and 6 h). Protein was determined by Western blot analysis. Each band density was normalized by its own ratio of p-MYPT/MYPT and results were expressed as fold induction respect to time 0. **B**: Means ± SD of at least three experiments was shown. * *P* < 0.05, when compared to time 0. # *P* < 0.05, when compared to HG alone.

**Figure 5:**
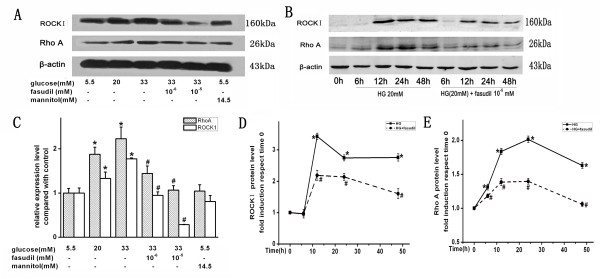
**Effects of HG and fasudil on RhoA and ROCKI protein in HUVECs.** RhoA and ROCKI protein was determined by Western blot analysis. Each band density was normalized by its own control. **A** is representative the dose-dependent effects of HG and fasudil. **B** is representative time-dependent effects of HG and fasudil. **C** is the summary of data (means ± SD) from the dose-dependent experiments. * *P* < 0.05 when compared to control. # *P* < 0.05 when compared to HG (33 mM) alone. **D** and **E** is the summary of data (means ± SD) from the time-dependent experiments. * *P* < 0.05 when compared to time 0, # *P* < 0.05, when compared to HG alone.

The ratio of phosphorylated MYPT1 to total MYPT1 is an index of the ROCK activity [20]. HG strongly increased p-MYPT/MYPT ratio, indicating that HG activates this pathway. As expected, fasudil reduced the p-MYPT/MYPT ratio (Figure 4). Interestingly, HG not only increased activity, but also increased expression of RhoA and ROCKI. This effect was also significantly attenuated by addition of fasudil at doses of 10^-6^ mM and 10^-5^ mM (Figure 5). These data demonstrated that fasudil prevented the HG induced increase both in Rho/ROCK expression and activity in endothelial cells.

### Effect of fasudil on sVCAM-1 and MCP-1 concentration in HUVECs supernatants

**Figure 6:**
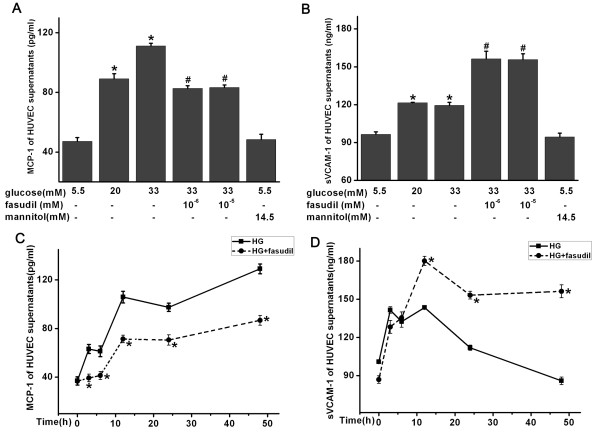
**Effect of fasudil on sVCAM-1 and MCP-1 of HUVECs supernatants.** sVCAM-1 and MCP-1 concentration in HUVECs supernatants were determined by ELISA. (A and B) Dose course of fasudil on concentration of MCP-1(**A**) and sVCAM-1(**B**) in HUVECs supernatants. * *P* < 0.05 when compared to control; # *P* < 0.05 when compared to HG alone. (C and D) Time course of fasudil and HG on concentration of MCP-1(**C**) and sVCAM-1(**D**) in HUVECs supernatants, * *P* < 0.05, when compared to HG alone.

Previous studies suggest that sVCAM-1 and MCP-1 are increased in patients with diabetes or coronary artery disease [21-23]. Therefore, the effects of fasudil on VCAM-1 and MCP-1 excretion by endothelial cells were studied. In HUVECs treated with HG (20 mM), a significant increase in release of sVCAM-1 and MCP-1 protein into the media was observed. This started at 3 h for both sVCAM-1 and MCP-1, and lasted for 12 h for sVCAM-1 and 48 h for MCP-1, respectively (Figure 6C and 6D). As we hypothesized, fasudil treatment significantly reduced soluble MCP-1 in the supernatants of HUVECs (*P* < 0.05) (Figure 6A and 6C). Interestingly, in contrast to MCP-1, sVCAM-1 concentration was elevated to a higher concentration after fasudil treatment (*P* < 0.05) (Figure 6B and 6D).

### Effect of fasudil on serum sVCAM-1 and MCP-1 in patients with diabetes

**Figure 7:**
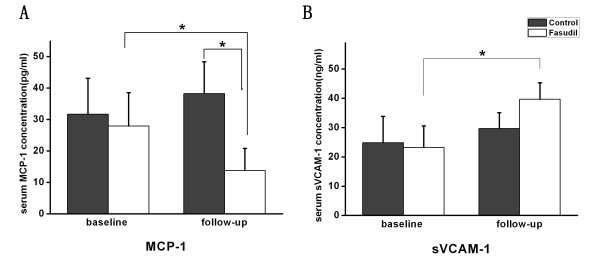
**Effect of fasudil on sVCAM-1 and MCP-1 in patients with diabetes.** sVCAM-1 and MCP-1 concentration in patients’ serum were determined by ELISA. Effect of fasudil intervention on serum concentration of MCP-1(**A**) and sVCAM-1 (**B**), * *P* < 0.05.

To extend our *in vitro* findings on the role of Rho/ROCK in expression of MCP-1 and VCAM-1, we examined the effects of fasudil on serum sVCAM-1 and MCP-1 levels in patients with diabetes. The basic characteristics of the diabetic patients with fasudil treatment are shown in Table 1. After administration of fasudil for 2 weeks, serum MCP-1 levels were decreased from 27.9 ± 10.6 pg/ml to 13.8 ± 7.0 pg/ml (*P* < 0.05), (Figure 7A). Consistent with our *in vitro* data, serum sVCAM-1 levels were increased from 23.2 ± 7.5 ng/ml to 39.7 ± 5.6 ng/ml after treatment with fasudil (*P* < 0.05) (Figure 7B). These data indicate that fasudil treatment can reduce soluble MCP-1 levels and increase sVCAM-1 levels in diabetic patients.

**Table 1:** Clinical and biochemical characteristics of the study population

**Parameter**	**Control (n = 10)**	**Fasudil (n = 10)**	***P* -Value**
Age, years	69 ± 9.4	71 ± 7.4	0.768
Male, n (%)	7 (70%)	6 (60%)	0.595
BMI, kg/m^2^	23.7 ± 3.0	25.3 ± 2.6	0.414
Smoking, n (%)	7 (70%)	8 (80%)	0.722
Hypertension, n (%)	6 (60%)	4 (40%)	0.357
Hyperlipidemia, n (%)	3 (30%)	2 (20%)	0.722
SBP, mmHg	138 ± 16.6	150.0 ± 25.5	0.427
DBP, mmHg	79 ± 8.5	86 ± 15.2	0.454
Total cholesterol, mmol/L	4.33 ± 0.87	4.48 ± 2.33	0.909
HDL-C, mmol/L	0.95 ± 0.27	1.09 ± 0.39	0.599
LDL-C, mmol/L	2.72 ± 0.49	2.84 ± 1.77	0.900
Triglycerides, mmol/L	1.46 ± 0.66	1.41 ± 0.39	0.906
FPG, mmol/L	5.58 ± 0.75	6.38 ± 1.87	0.467
hsCRP, mg/L	6.85 ± 4.08	6.37 ± 4.78	0.437
Alanine aminotransferase, U/L	29.5 ± 13.31	26.5 ± 5.69	0.865
BUN, mmol/L	6.30 ± 0.53	5.90 ± 2.44	0.756

Continuous variables were described as means ± SD; categorical variables were presented as frequencies. BMI, body mass index; SBP, systolic blood pressure; DBP, diastolic blood pressure; n, number of patients; LDL-C, low-density lipoprotein cholesterol; HDL-C, high-density lipoprotein cholesterol; FPG, fasting plasma glucose; BUN, blood urine nitrogen; hsCRP, high-sensitivity CRP.

## Discussion

The present study demonstrated that ROCK inhibitor fasudil attenuated HG-induced increases in VCAM-1 and MCP-1 expression in HUVECs. In addition, fasudil decreased MCP-1 secretion and increased sVCAM-1 secretion both *in vitro* and *in vivo*. Our data suggested that fasudil may protect vessels from monocyte adhesion by both decreasing VCAM-1 expression and by increasing shedding (increasing sVCAM-1).

Atherosclerosis is a chronic inflammatory process. One of the earliest events in the vascular inflammation process is the adhesion of monocytes to the endothelium, followed by their infiltration and differentiation into macrophages [2,3]. This key step is mediated by the interaction between monocytes and the molecules expressed on the endothelial cell surface [24,25]. Hyperglycemia is able to influence on these events. In endothelial cells, hyperglycemia decrease nitric oxide, and increase oxidative stress and receptor for advanced glycation end products activation [26]. Those factors will increase NF-κB activation which upregulate chemoattractant and adhesion molecules expression [27,28], that recruit lymphocytes and monocytes into the vascular wall and facilitate the monocyte-endothelial adhesion and monocytes transendothelial migration. Monocytes, upon reaching the subendothelial space, ingest oxidized low density lipoprotein via scavenger receptors and become foam cells. Localized accumulation of foam cells lead to formation of fatty streaks, the hallmark of early atherosclerotic lesions.

Rho and its target ROCK have been reported to play a crucial role in cardiovascular disease. In vascular smooth muscle differentiation, the RhoA/ROCK signaling pathway provides an important activating stimulus for MEF2C mediated induction of myocardin expression in VSMCs, through a mechanism involving p38 MAPK, protein phosphatase 1α, and CPI-17 [29]. In cardiovascular hypertrophy, ROCK activation promotes inflammation and angiotensin II-induced remodeling through promoting ROS production through upregulation of NADPH oxidases [30]. In ischemic heart disease, RhoA/ROCK inhibitor fasudil induces postconditioning against myocardial infarction via m-KATP channels in the rat; although hyperglycemia attenuates it, high-dose fasudil can restore cardioprotection [31].

Recent studies also found that RhoA/ROCK-dependent moesin phosphorylation involved in the advanced glycation end products-mediated endothelial dysfunction [32]. Rho/ROCK pathway activation in endothelial cells and leucocytes participates in the regulation of genes such as intercellular adhesion molecule-1 [33], angiotensin II-induced MCP-1 and plasminogen activator inhibitor-1 [34,35]. Previous studies suggest that Rho/ROCK signal pathway modulates gene expression via regulating the serum response factor activity [36,37] or other signal pathway such as ERK [38], p38 MAPK, CPI-17, and so on [29]. Furthermore, treatments with fasudil caused a decrease in arterialintima-medial thickness, maximum flow velocity and macrophage accumulation in the atherosclerosis lesions [12]. Fasudil also showed similar effects of exercise on plaque morphology and composition [39].

In our study, VCAM-1 and MCP-1 expression in HUVECs was significantly increased in a dose- and time-dependent manner in response to HG. Our findings suggested that the increase in VCAM-1 and MCP-1 levels induced by HG was dependent on Rho/ROCK pathway, since the expression of VCAM-1, MCP-1 proteins and adhesion of monocytes to HUVECs were significantly suppressed when the HUVECs were treated with fasudil. Moreover, we found that the effect of HG on Rho/ROCK signaling was not only through up-regulation of Rho/ROCK pathway activity, but also through increased expression of Rho/ROCK protein. From these observations, we suggested that HG may have both short term and long term effects on vessel function, through acute increase of Rho/ROCK activity, as well as through long term upregulation of Rho and ROCK protein expression.

In this study, fasudil decreased mRNA, protein expression and serum protein levels of MCP-1 both *in vivo and in vitro*. Interestingly, the case of sVCAM-1 was different from that of MCP-1. In cultured HUVECs, sVCAM-1 was increased in response to HG, but even further elevated in response to fasudil. Consistent with our *in vitro* studies, increased serum levels of sVCAM-1 were also observed in patients with diabetes after fasudil treatment. VCAM-1 has a molecular structure resembling that of immunoglobulin and facilitates endothelial adhesion of circulating leukocytes, including lymphocytes and monocytes, through binding the very late antigen-4, which is expressed on the surface of these cells [40]. VCAM-1 can be cleaved to sVCAM-1 by disintegrin and metalloproteinase 17 (ADAM-17) [41]. Cleavage of VCAM-1 (namely sVCAM-1) is predicted to affect its function at several levels [41]: First, cleavage of VCAM-1 may regulate the adhesive function of VCAM-1 by decreasing its levels at the cell surface; a second potential implication of VCAM-1 shedding is that soluble ectodomain may remain functionally active to bind to leukocytes and block adhesion to VCAM-1 on the endothelial cells. Previous report also suggests that sVCAM-1 is a sensitive marker of endothelial activation [42] and increases in the levels of soluble adhesion molecules correlate with a variety of inflammatory diseases. For example, studies show that sVCAM-1 can increase in patients with diabetes or coronary artery disease (including acute coronary syndromes) [21,22,43].

In addition to our observation of an increase in soluble VCAM-1 in response to Rho/ROCK inhibition, a previous study showed that cerivastatin could also increase sVCAM-1 shedding in HUVECs, and this was reversed by mevalonate and nonsteroidal isoprenoids [44]. It is believed that, some of the beneficial effects of statins may result from their effects on the RhoA/ROCK pathway. Statins decrease the synthesis of isoprenoids, thus inhibiting RhoA geranylgeranylation and reducing membrane GTP-bound active RhoA and subsequent ROCK activity [10,45]. Our data demonstrate that direct inhibition of Rho/ROCK also increases soluble VCAM-1 levels, suggesting a potential mechanism for increased sVCAM-1 in response to statins.

Diabetes may lead to early-onset vascular impairment; however, to date, treatment for this aspect of diabetes is very limited. Our studies indicated that the inhibition of Rho/ROCK pathway shows great potential as a protection against diabetic vascular complications by inhibiting the hyperglycemia-induced vascular inflammatory process in vessels.

## Limitations

The current study had some limitations which should be taken into account. First, the number of patients enrolled in our study was relatively small and none of the subjects had macrovascular complications. Thus the clinical value of fasudil for diabetes needs to be further studied. Second, the patients were treated with fasudil intravenously for only two weeks, so the long term effects of fasudil were not determined in our study. This will be the subject of a future investigation.

## Conclusions

Taken together, our results indicate that ROCK inhibitor fasudil attenuate high glucose-induced monocyte adhesion to endothelial cells, ostensibly through limiting expression of endothelial VCAM-1 and MCP-1. In addition, fasudil attenuate HG-induced MCP-1 excretion by endothelial cells, while increasing release of sVCAM-1. These data suggest that fasudil may protect against vascular inflammation in diabetes, in part by limiting expression of monocyte chemotactic and adhesion factors by endothelial cells.

## Abbreviations

eNOS: Endothelial nitric oxide synthase; HG: High glucose; HUVECs: Human umbilical vein endothelial cells; MCP-1: Monocyte chemoattractant protein-1; mM: mmol/L; ROCK: Rho kinase; PBS: Phosphate-buffered saline; VCAM-1: Vascular cell adhesion molecule-1; sVCAM-1 Soluble vascular cell adhesion molecule-1.

## Competing interest

The authors had no conflicts of interest to declare in relation to this article.

## Authors’ contributions

XYW and PWH designed, coordinated and wrote the manuscript. LHL coordinated and wrote the manuscript, performed cell culture, RT-PCR and western blot. JWX, LYM and LWM carried out the sample collection. JWX performed ELISA. LQ and LHL performed statistical analysis. All authors read and approved the final manuscript.
